# Deletion of the nuclear receptor RORα in macrophages does not modify the development of obesity, insulin resistance and NASH

**DOI:** 10.1038/s41598-020-77858-6

**Published:** 2020-12-03

**Authors:** Laurent L’homme, Benan Pelin Sermikli, Olivier Molendi-Coste, Sébastien Fleury, Sandrine Quemener, Mathilde Le Maître, Marie-Laure Joseph, Laurent Pineau, Christian Duhem, Barbara Gross, Emmanuelle Vallez, Anne Tailleux, Bart Staels, David Dombrowicz

**Affiliations:** Univ. Lille, Inserm, CHU Lille, Institut Pasteur de Lille, U1011-EGID, 59000 Lille, France

**Keywords:** Monocytes and macrophages, Kupffer cells, Non-alcoholic fatty liver disease, Non-alcoholic steatohepatitis, Obesity, Type 2 diabetes

## Abstract

Retinoic acid receptor-related orphan receptor-alpha (RORα) is a transcription factor from the nuclear receptor family expressed by immune cells and involved in the development of obesity, insulin resistance (IR) and non-alcoholic steatohepatitis (NASH). It was recently reported that mice deficient for RORα in macrophages develop more severe NASH upon high fat diet (HFD) feeding due to altered Kupffer cell function. To better understand the role of RORα in obesity and IR, we independently generated a macrophage RORα-deficient mouse line. We report that RORα deletion in macrophages does not impact on HFD-induced obesity and IR. Surprisingly, we did not confirm an effect on NASH development upon HFD feeding nor in the more severe and obesity-independent choline-deficient, L-amino acid-defined diet model. Our results therefore show that RORα deletion in macrophages does not alter the development of obesity and IR and question its role in NASH.

## Introduction

During the last few decades, the prevalence of obesity has dramatically increased worldwide, arising from excessive dietary intake and a sedentary lifestyle^1^. Obesity is a strong risk factor for type 2 diabetes (T2D) and non-alcoholic fatty liver disease (NAFLD). T2D is characterized by hyperglycemia resulting from insulin resistance (IR) and relative insulin deficiency^2^. The chronic and low-grade systemic inflammation developing during obesity is considered as one critical step in the pathogenesis of IR and represents a valuable therapeutic target^3^. In obesity, enlarged adipocytes promote the recruitment and activation of immune cells leading to the accumulation of monocytes, pro-inflammatory macrophages, neutrophils, T cells and B cells in adipose tissue (AT)^4^. AT macrophages (ATM) are the most abundant leukocytes in obese AT and among the most important immune cell types mediating inflammation and IR^5,6^. ATM secrete several pro-inflammatory cytokines such as IL-1β, TNF-α and IL-6, all interfering with the insulin signaling pathway^7^. Expansion of ATM mainly results from tissue recruitment of circulating monocytes followed by their differentiation into pro-inflammatory macrophages^8^.

NAFLD comprises a liver phenotype spectrum ranging from simple steatosis, also called non-alcoholic fatty liver (NAFL), to non-alcoholic steatohepatitis (NASH). NAFLD diagnosis is based on histology with steatosis, characterized by triglyceride accumulation in hepatocytes, being the first stage^9^. NAFL progresses into NASH with the appearance of lobular inflammation and hepatocyte ballooning. NASH predisposes to fibrosis and may ultimately evolve into cirrhosis and hepatocellular carcinoma^10^. NAFLD is strongly associated with obesity, IR and the capacity of AT to store lipids^11^. The AT-liver crosstalk is essential in NAFLD pathophysiology. Indeed, development of IR in AT increases free fatty acids (FFAs) release, leading to FFAs uptake and esterification into triglycerides in the liver. Excessive lipid accumulation within hepatocytes induces lipotoxicity and the release of damage-associated molecular patterns (DAMPs) that ultimately activate immune cells and favor leukocyte recruitment. Immune cells, and particularly the resident liver macrophages (Kupffer cells, KCs), play key roles in NAFLD by regulating hepatocyte metabolism, inflammation and fibrosis^12,13^.

These metabolic and inflammatory processes are tightly regulated at the transcriptional level by a complex network of interactions. Among them, transcription factors such as nuclear receptors play prominent roles. The retinoic acid receptor-related orphan receptors (RORs) family consists of RORα (NR1F1), RORβ (NR1F2), and RORγ (NR1F3). RORs have the common nuclear receptor structure with a variable N-terminal A/B domain, a two zinc fingers-containing DNA-binding domain (DBD), a hinge region and a C-terminal ligand-binding domain (LBD). The DBD is highly conserved between RORs and bind the same ROR response element (RORE). RORα and RORγ are widely expressed, whereas RORβ is more restricted to the central nervous system^14^. The RORα gene (*RORA*) produces four different isoforms in humans (RORα1-4) and two in mice (RORα1 and RORα4). RORα1 and RORα4 are generated by alternative promoter usage and diverge only in the N-terminal A/B domain. RORα regulates the expression of genes involved in lipid and glucose metabolism, circadian rhythm and immunity^15^. It has been shown that RORα regulates the development and function of several immune cell types, such as group 2 innate lymphoid cells^16^, Th17 cells^17^, IgA-producing B cells^18^ and macrophages^19^.

A growing body of evidence also suggests a role for RORα in obesity, T2D and NAFLD. In humans, mutations in the RORA gene is associated with obesity^20^, T2D^21^ and liver diseases^22^. The staggerer mice (*Rora*^*sg/sg*^), which are carrying a natural mutation in the RORα LBD, are protected against diet-induced obesity, IR and NAFLD^23,24^. However, due to the high expression of RORα in the cerebellum and its critical role in the development of Purkinje cells, *Rora*^*sg/sg*^ mice exhibit a severe cerebellar ataxia, motor defect and tremor. *Rora*^*sg/sg*^ also display increased energy expenditure which likely contributes to the protection against obesity^23^. Concomitantly, the development of new synthetic ligands also provided evidences for a role of RORα in IR and NAFLD^25,26^. Recently, hepatocyte-specific RORα deletion was reported to exacerbate NASH development^27,28^. However, in an independent study, no effect of hepatocyte-specific RORα deletion on NASH was observed^29^, leaving open the question about how RORα influences NASH. Since immune cells, and particularly macrophages, are important in the development of obesity-related diseases including T2D and NASH, we hypothesized that the role of RORα in these pathologies might in part result from its expression in macrophages. In the present work, we therefore generated a mouse line harboring RORα deletion in macrophages and evaluated its impact in models of obesity, IR and NASH.

## Results

### Characterization of RORα MKO mice

**Figure 1:**
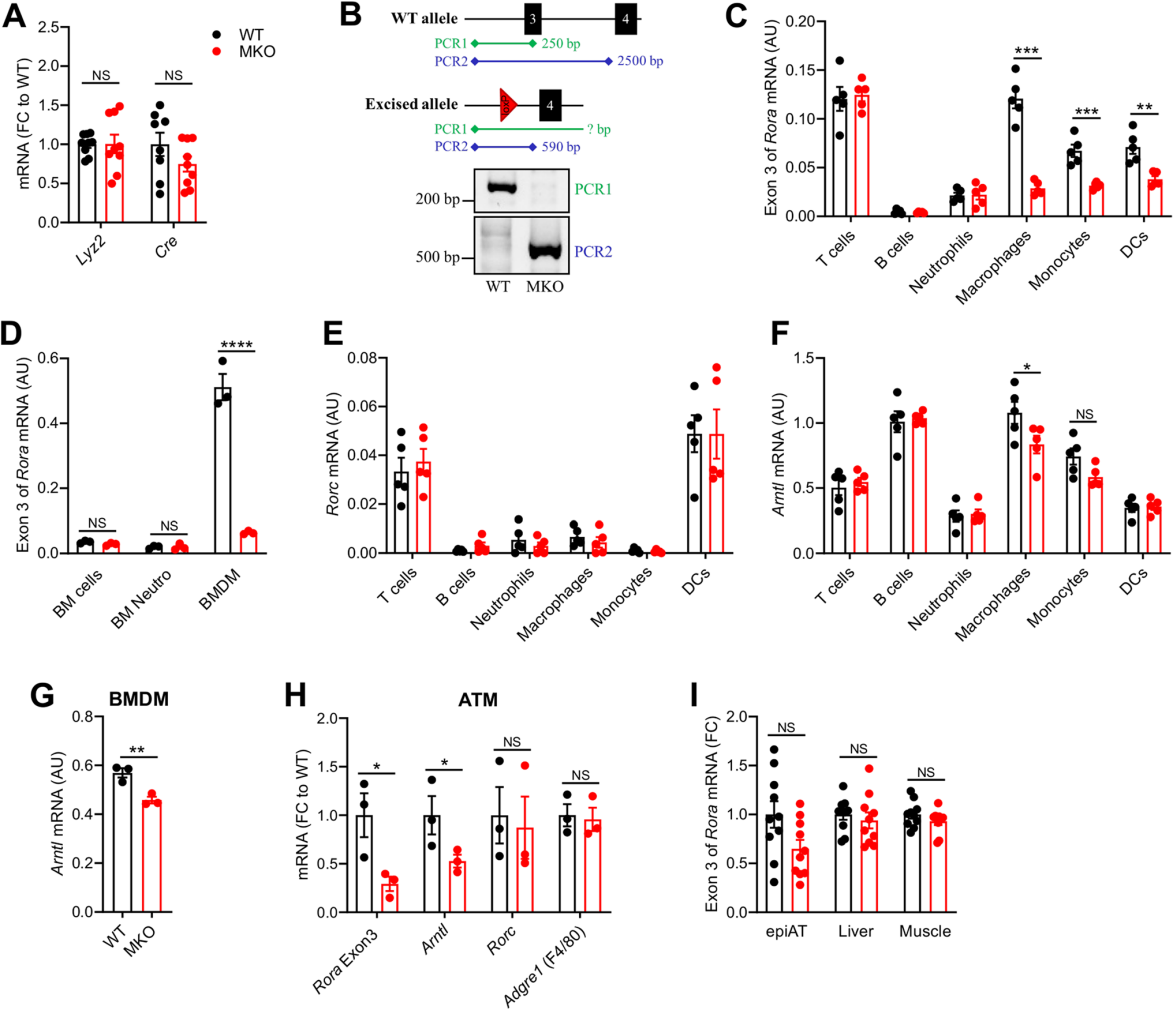
Characterization of RORα MKO mice. (**A**) mRNA expression levels measured by RT-qPCR for *Lyz2* and *Cre* genes in spleen from WT (*Rora*^+/+^ *Lyz2*^Cre/+^) and MKO (*Rora*^fl/fl^ *Lyz2*^Cre/+^) mice. (**B**) PCR analysis of genomic DNA extracted from sorted splenic macrophages of WT and MKO mice. Locations of primer hybridization are represented with filled diamond. Agarose gels were acquired with a Gel Doc XR system (Bio-Rad) and the Image Lab software verion 2.0 build 8 for PC (Bio-Rad, https://www.bio-rad.com/fr-fr/product/image-lab-software?ID=KRE6P5E8Z). Images were cropped for sake of clarity and full-length gels are presented in Supplementary Fig. 6. (**C**) mRNA expression levels measured by RT-qPCR for the exon 3 of *Rora* in B cells, T cells, neutrophils, macrophages, monocytes, and dendritic cells (DCs) sorted from the spleen of WT and MKO mice. (**D**) mRNA expression levels measured by RT-qPCR for the exon 3 of *Rora* in total bone marrow (BM) cells, in neutrophils purified from BM and in BM-derived macrophages (BMDM) from WT and MKO mice. (**E**,**F**) mRNA expression levels measured by RT-qPCR for *Rorc* (**D**) and *Arntl* (**E**) genes in B cells, T cells, neutrophils, macrophages, monocytes, and DCs sorted from the spleen of WT and MKO mice. (**G**) mRNA expression levels measured by RT-qPCR for *Arntl* in BMDM. (**H**) mRNA expression levels measured by RT-qPCR for the exon 3 of *Rora*, *Arntl*, *Rorc* and *Adgre1* in adipose tissue macrophages (ATM) sorted from epididymal adipose tissue of WT and MKO mice. (**I**) mRNA expression levels measured by RT-qPCR for the exon 3 of *Rora* in liver, epididymal adipose tissue (epiAT) and skeletal muscle from WT and MKO mice. Data are shown as mean ± SEM. * *p* < 0.05; ** *p* < 0.01; *** *p* < 0.001 by 2-way ANOVA followed by Sidak’s multiple comparisons test or unpaired t test. All statistical analyses were carried out using GraphPad Prism 8 for Windows (GraphPad Software). n = 8–10 mice per genotype for **A** & **I**. n = 5 mice per genotype for **C**,**E** & **F**. n = 3 mice per genotype for **D** & **G**. For **H**, each dot represents a pool of 3–4 mice. NS: Not significant; AU: Arbitrary unit; FC: Fold change. See also Fig. S1.

We generated *Rora* floxed mice by introducing loxP sites flanking exon 3 (Fig. S1A). Exon 3, the first common exon of isoforms 1 and 4, encodes for the DBD and includes the first zinc finger motif. Deletion of exon 3 leads to a frameshift resulting in a premature stop codon and the absence of functional domains (Fig. S1B,C). To achieve RORα deletion in macrophages, these mice were subsequently crossed with the LysM-Cre mice. *Rora*^*fl/fl*^* Lyz2*^*Cre/*+^ (MKO) mice and their littermate controls *Rora*^+*/*+^ *Lyz2*^*Cre/*+^ (WT), exhibiting comparable expression of *Lyz2* and *Cre* (Fig. 1A), were further studied.

To validate and characterize the RORα deletion in MKO mice, we demonstrated the excision of exon 3 in DNA from sorted splenic macrophages (Fig. 1B). Since the LysM-Cre mouse model mainly targets macrophages and neutrophils, but also a fraction of monocytes and DCs^30^, we sorted these populations from spleen and quantified the deletion of exon 3 in *Rora* mRNA by RT-qPCR (Fig. 1C). MKO mice displayed an important deletion of exon 3 in macrophages and to a lesser extent in monocytes and DCs, but surprisingly not in neutrophils. No deletion of exon 3 in *Rora* mRNA was observed in T cells and B cells. To confirm the absence of deletion in neutrophils, we also analyzed the bone marrow (BM) cells which mainly contain neutrophils. Neither total BM cells nor purified neutrophils from BM showed a deletion of exon 3, while bone marrow-derived macrophages (BMDM) did (Fig. 1D). Collectively, our results show that macrophages, monocytes and DCs, but not neutrophils, exhibit a LyzM-Cre-induced deletion of *Rora* exon 3.

*Rorc* expression was not affected by *Rora* deletion (Fig. 1E) and *Rorb* was undetectable in both WT and MKO immune cells (data not shown), suggesting no compensatory regulation of other ROR genes. The expression of a key ROR target gene *Arntl*, encoding for BMAL1, was significantly lower in RORα-deficient splenic macrophages and BMDM (Fig. 1F,G). A tendency to a lower *Arntl* expression was also observed in monocytes, but not in DCs, probably due to the high expression of *Rorc* (Fig. 1E). Thus, despite the significant LysM-Cre-induced deletion of exon 3 in monocytes and DCs, MKO mice foremost displayed a phenotype in macrophages. As macrophages display some tissue-specific characteristics, we sorted ATM (CD45^+^ CD3ε^−^ TCRβ^−^ CD19^−^ CD20^−^ F4/80^+^ CD64^+^) from epididymal AT to evaluate the efficiency of *Rora* deletion in these cells. Similar to the splenic macrophages and BMDM, MKO mice showed a significant deletion of exon 3 and a significant decrease of the ROR target gene *Arntl* in ATM (Fig. 1H). No significant deletion of exon 3 occurred in total epididymal AT, liver nor skeletal muscle (Fig. 1I), suggesting the absence of an unwanted recombination in parenchymal cells of metabolic tissues.

### RORα deletion in macrophages does not affect obesity, IR nor steatosis

**Figure 2:**
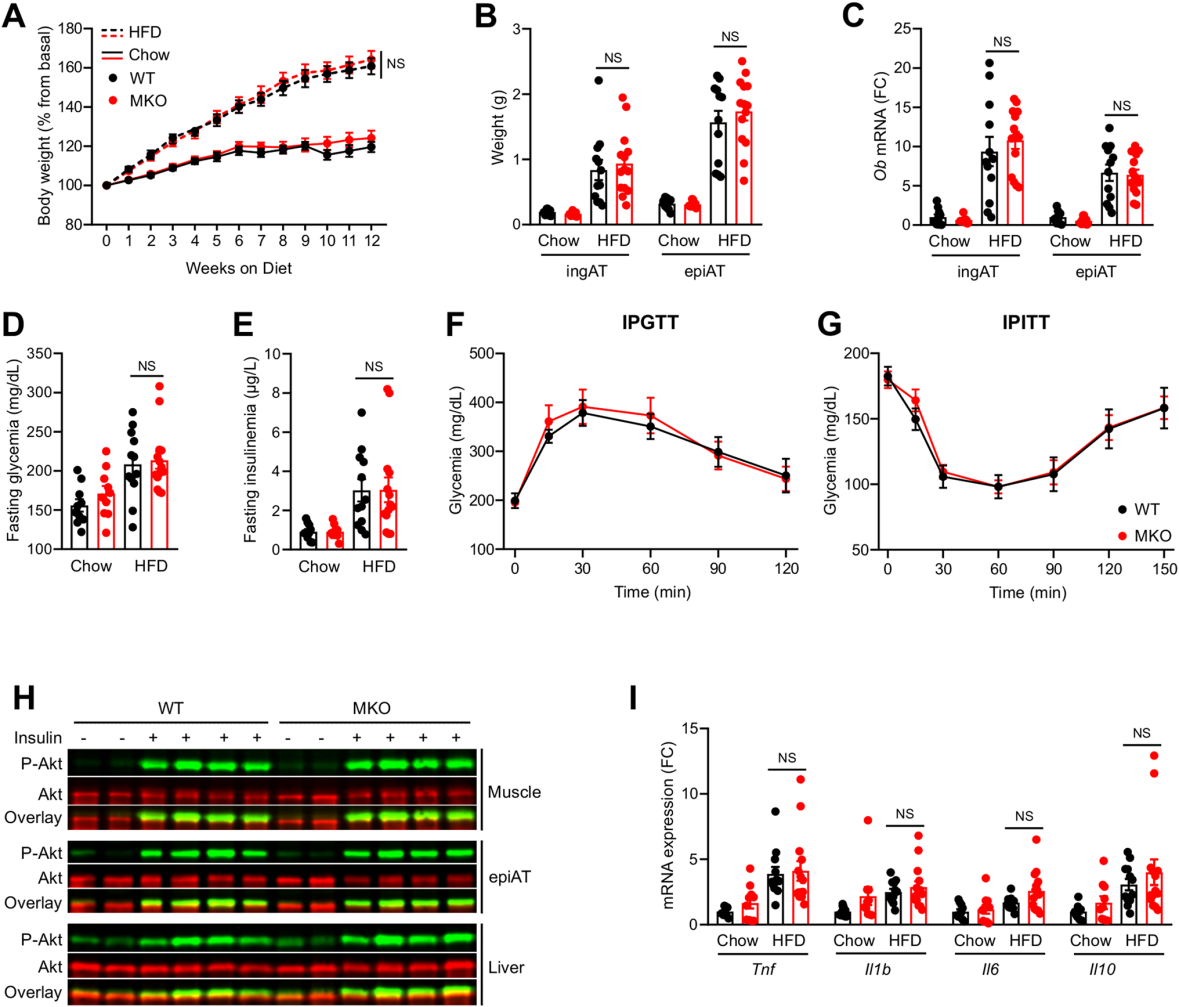
Effect of RORα deletion in macrophages on HFD-induced obesity and IR. Ten weeks old WT and MKO mice were fed with either a chow or a HFD for 12 weeks. (**A**) Body weight gain. (**B**) Weight of inguinal (ingAT) and epidydimal (epiAT) adipose tissues. (**C**) mRNA expression levels measured by RT-qPCR for the *Ob* gene in both ingAT and epiAT. (**D**,**F**) After 10 weeks of diet, mice were fasted for 5 h and glycemia (**D**) and insulinemia (**E**) were measured, then intraperitonealy injected with 1 g/kg glucose for a glucose tolerance test (IPGTT) (**F**). (**G**) Intraperitoneal insulin tolerance test (IPITT) was performed by injecting 1 IU/kg insulin after 11 weeks of diet and 5 h of fasting. (**H**) Western blot of phospho-AKT^Ser473^ in skeletal muscle, epiAT and liver. Mice were injected with either PBS or 1 IU of insulin 15 min before sacrifice. Western blots were scanned with an Odyssey CLx Imaging System (LI-COR) and with the Image studio Version 4.0.21 software (LI-COR, https://www.licor.com/bio/image-studio/). Images were cropped for sake of clarity and full-length blots are presented in Supplementary Figs. 7 to 9. (**I**) mRNA expression levels measured by RT-qPCR for *Tnf*, *Il1b*, *Il6*, and *Il10* genes in epiAT. Data are shown as mean ± SEM. 2-way ANOVA followed by Sidak’s multiple comparisons test was performed. All statistical analyses were carried out using GraphPad Prism 8 for Windows (GraphPad Software). n = 10–15 mice per group. NS: Not significant; FC: Fold Change. See also Fig.S2.

To investigate the role of macrophages RORα in metabolic functions, in particular obesity and IR, we fed WT and MKO mice with a 60% high fat diet (HFD) for 12 weeks. WT and MKO mice under HFD significantly gained weight, AT mass and increased leptin expression similarly in both genotypes (Fig. 2A–C). Fasting serum glucose and insulin concentrations increased significantly upon HFD feeding although no difference between genotypes was observed (Fig. 2D,E). Furthermore, we observed no difference in glucose levels between WT and MKO mice during IPGTT and IPITT (Fig. 2F,G). Moreover, insulin signaling in skeletal muscle, epididymal AT and liver was similar in both genotypes (Fig. 2H and S2A). AT inflammation is a central driver for obesity-induced IR^4^. No difference in *Tnf*, *Il1b*, *Il6*, and *Il10* expression was observed in epididymal AT between WT and MKO mice (Fig. 2I). Likewise, plasma cholesterol and triglyceride levels were not different between WT and MKO mice (Fig. S2B,C). Taken together, our results show that RORα deletion in macrophages has no impact on HFD-induced obesity and IR.

**Figure 3:**
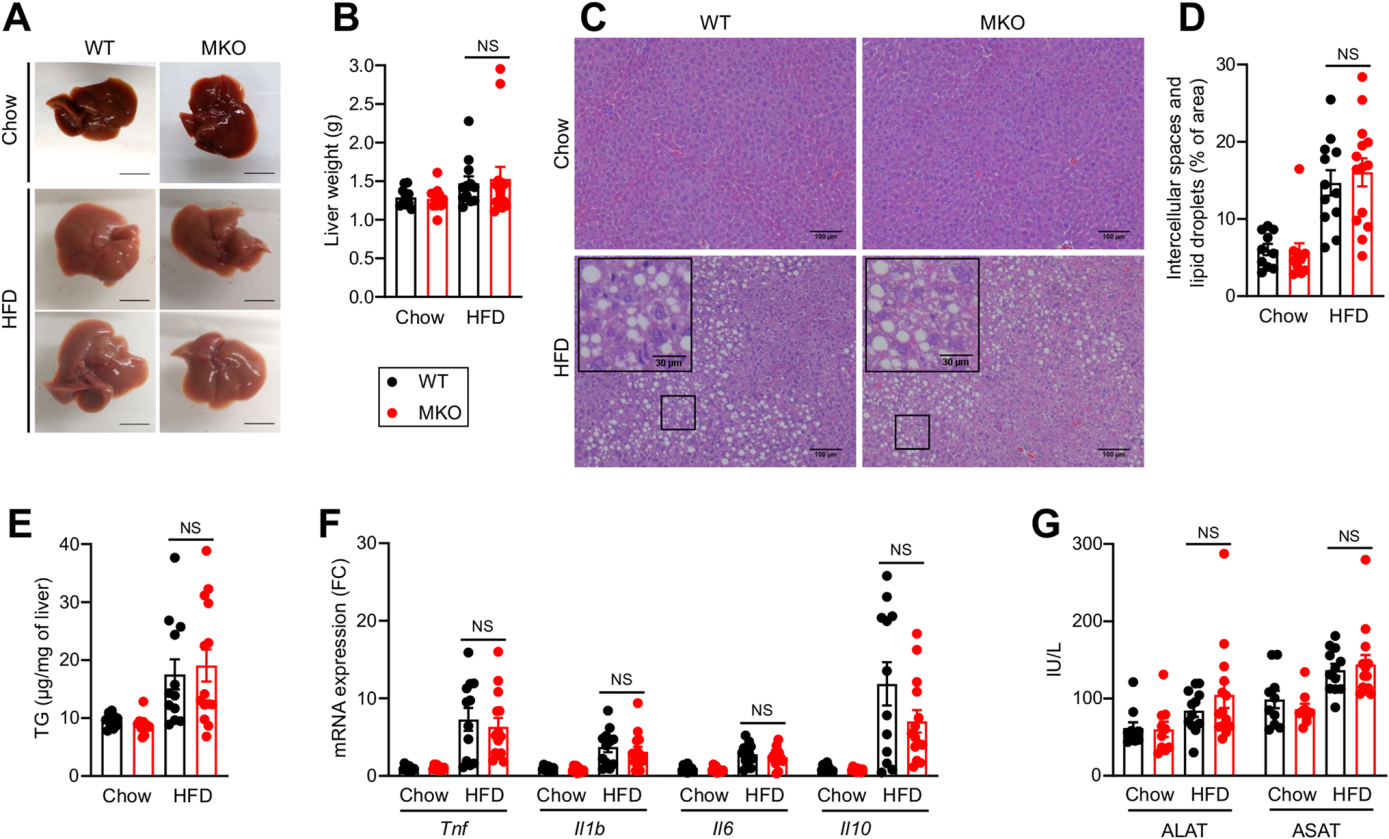
Effect of RORα deletion in macrophages on liver upon HFD feeding. Ten weeks old WT and MKO mice were fed with either a chow or a HFD for 12 weeks. (**A**) Representative liver gross morphology. (**B**) Liver weight. (**C**, **D**) Hematoxylin & Eosin staining of liver sections (**C**) and quantification of lipid droplets (**D**). Images were acquired on an Eclipse Ti-U microscope (Nikon) and quantified with Image J version 1.51j8 software (NIH, https://imagej.nih.gov/ij/). (**E**) Hepatic triglycerides (TG) content. (**F**) mRNA expression levels measured by RT-qPCR for *Tnf*, *Il1b*, *Il6*, and *Il10* genes in liver. (**G**) Plasma transaminases activity. Data are shown as mean ± SEM. 2-way ANOVA followed by Sidak’s multiple comparisons test was performed. All statistical analyses were carried out using GraphPad Prism 8 for Windows (GraphPad Software). n = 10–15 mice per group. NS: Not significant; FC: Fold Change. See also Fig. S3.

In interconnection with obesity and IR, HFD feeding also induces liver steatosis. Liver weight, steatosis level determined by histology and hepatic triglyceride content were increased upon HFD feeding to the same extent in WT and MKO mice (Fig. 3A–E). Histology analysis did not reveal cell infiltrates upon HFD feeding despite the significantly increased expression of inflammatory genes such as *Tnf*, *Il1b*, *Il6* and *Il10* (Fig. 3F). Likewise, we did not observe any significant differences in hepatic cytokine expression between WT and MKO mice besides a tendency to a lower *Il10* expression in MKO mice (*p* = 0.07). HFD feeding induced a similar mild increase of plasma transaminase activity in both WT and MKO mice (Fig. 3G). Liver fibrosis and expression of collagen genes slightly increased upon HFD feeding for 12 weeks at comparable level between WT and MKO mice (Fig. S3A–C).

### RORα deletion in macrophages does not affect NASH

**Figure 4:**
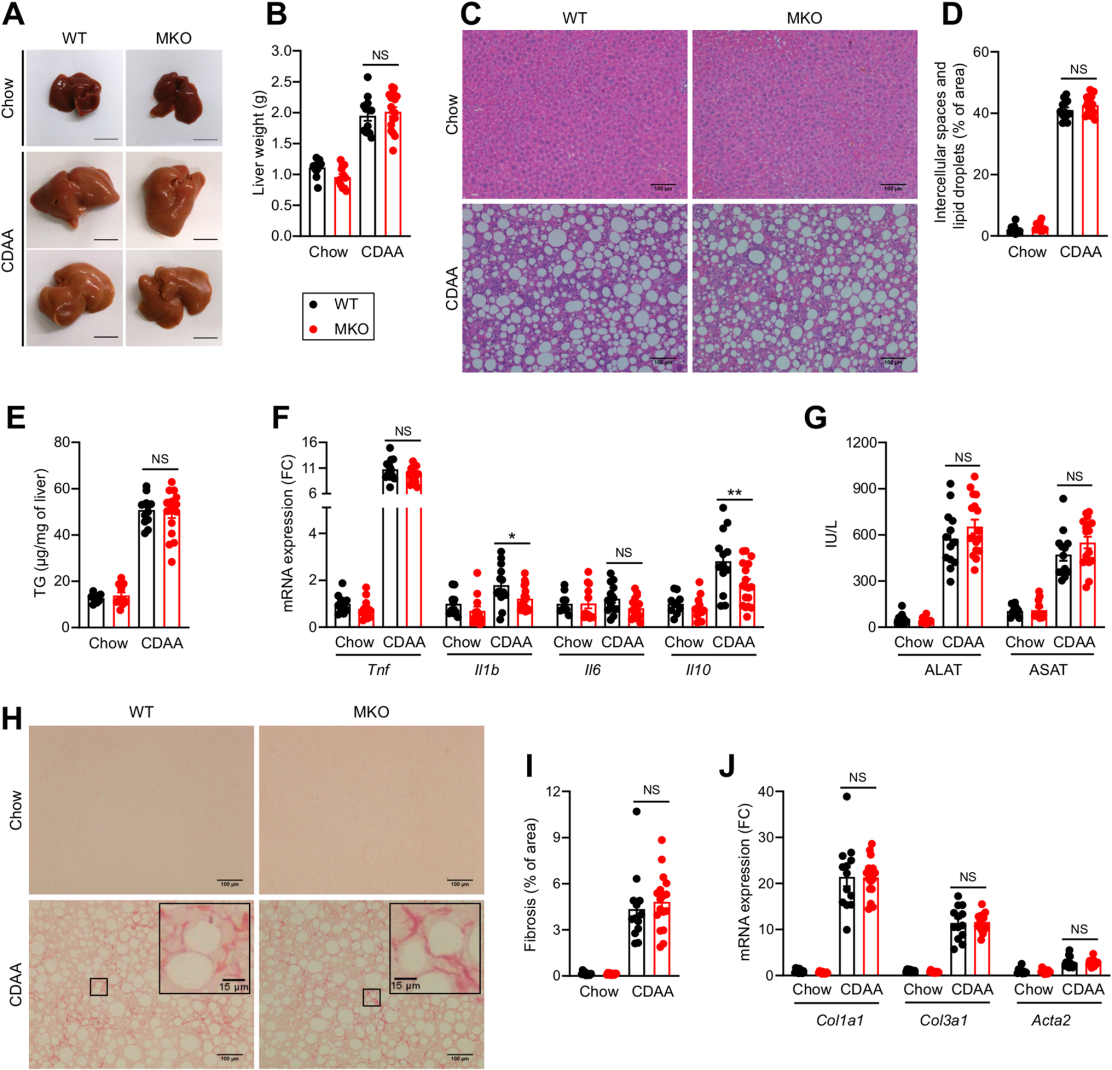
Effect of RORα deletion in macrophages on liver upon CDAA diet feeding. Ten weeks old WT and MKO mice were fed with either a chow or a CDAA diet for 8 weeks. (**A**) Representative liver gross morphology. (**B**) Liver weight. (**C**,**D**) Hematoxylin & Eosin staining of liver sections (**C**) and quantification of lipid droplets (**D**). Images were acquired on an Eclipse Ti-U microscope (Nikon) and quantified with Image J version 1.51j8 software (NIH, https://imagej.nih.gov/ij/). (**E**) Hepatic triglycerides (TG) content. (**F**) mRNA expression levels measured by RT-qPCR for *Tnf*, *Il1b*, *Il6*, and *Il10* genes in liver. (**G**) Plasma transaminases activity. (**H**,**I**) Sirius red staining of liver sections (**H**) and quantification of fibrosis (**I**). Images were acquired on an Eclipse Ti-U microscope (Nikon) and quantified with Image J version 1.51j8 software (NIH, https://imagej.nih.gov/ij/). (**J**) mRNA expression levels measured by RT-qPCR for *Col1a1*, *Col3a1*, and *Acta2* genes in the liver. Data are shown as mean ± SEM. *p < 0.05 by 2-way ANOVA followed by Sidak’s multiple comparisons test. All statistical analyses were carried out using GraphPad Prism 8 for Windows (GraphPad Software). n = 10–17 mice per group. NS: Not significant; FC: Fold Change. See also Fig. S4.

While HFD feeding efficiently promotes obesity and IR, the liver pathology is mainly characterized by triglyceride accumulation corresponding to the NAFL stage^9^. To investigate whether the deletion of RORα in macrophages might play a role in advanced NAFLD, WT and MKO mice were fed with the choline-deficient, L-amino acid-defined (CDAA) diet supplemented with sucrose and 2% cholesterol for 8 weeks, leading to a pronounced hepatic steatosis, inflammation and fibrosis, but dissociated from obesity and IR allowing the analysis of liver-centric responses^9^. As expected and unlike HFD, the CDAA diet did not induce obesity, hyperglycemia nor AT inflammation, but led to a 10% weight loss and a drop of glycemia of 30% in both WT and MKO (Fig. S4A–C). The CDAA diet produced a massive hepatic steatosis, but RORα deletion in macrophages did not impact on liver weight nor steatosis level (Fig. 4A–E). Histological analysis showed evidences of cell infiltrates upon CDAA diet feeding but no manifest difference between WT and MKO mice was observed (Fig. 4C & S4D). Further analysis of the expression of inflammatory genes revealed no difference in *Tnf* and *Il6* expression whereas *Il1b* and *Il10* mRNA significantly decreased in livers of MKO mice (Fig. 4F). Despite this modest decrease in cytokine expression, we did not observe any significant differences in plasma transaminase activity nor fibrosis, while a strong effect of the CDAA diet was found on these parameters (Fig. 4G–J & S4E,F). These results indicate that deletion of RORα in macrophages does not affect CDAA-induced NASH.

### Effect of RORα deletion in KC is offset by high RORγ expression

**Figure 5:**
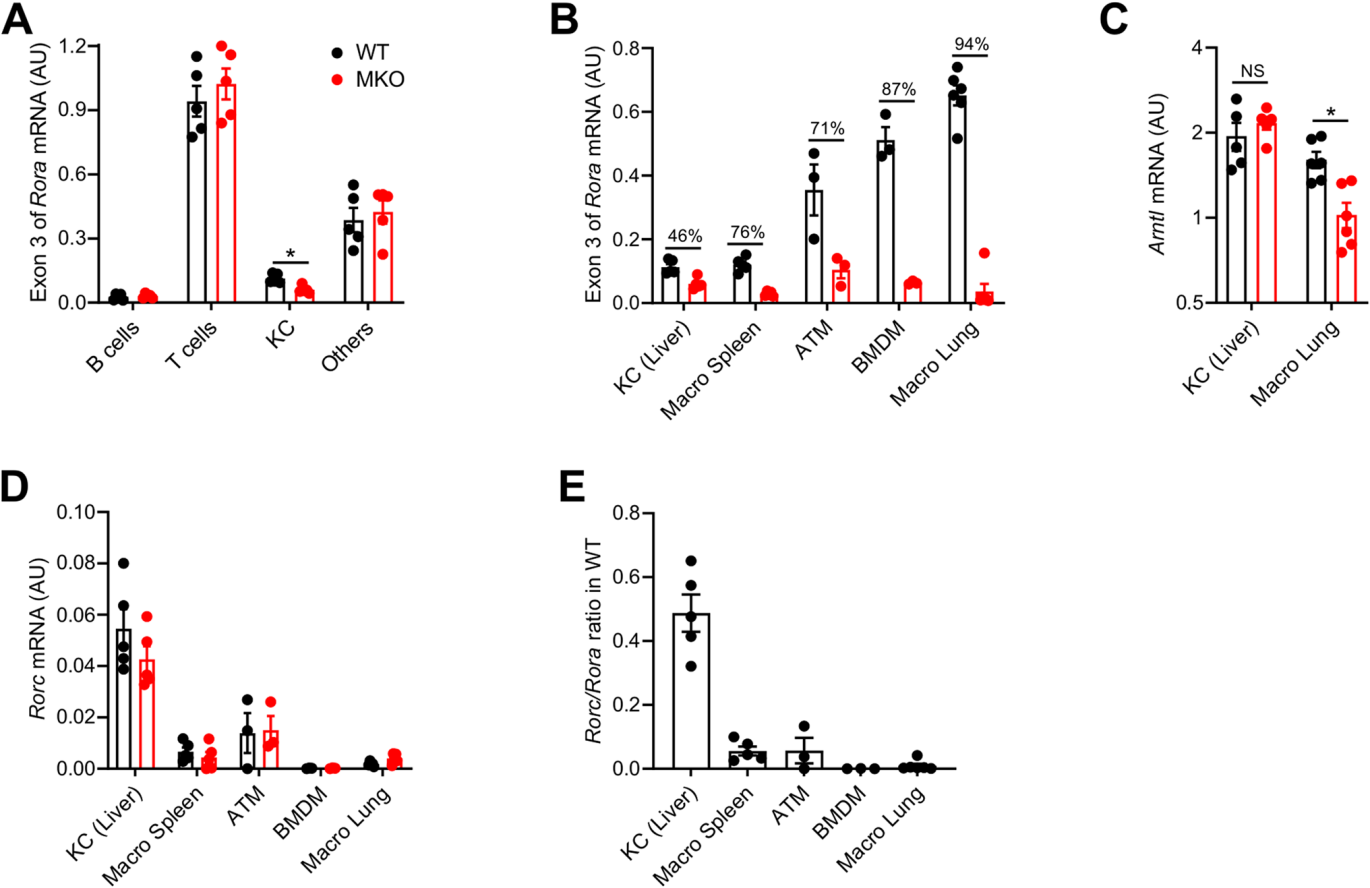
Impact of RORα deletion on Kupffer Cells (KCs). (**A**,**B**) mRNA expression levels measured by RT-qPCR for the exon 3 of *Rora* gene in B cells, T cells, KCs, and CD45^+^ non T, B or KC (others) sorted from liver (**A**) and in different macrophage populations (**B**) from WT and MKO mice fed with chow diet. (**C**,**D**) mRNA expression levels measured by RT-qPCR for *Arntl* (**C**) and *Rorc* (**D**) genes in different macrophage populations. (**E**) *Rorc*/*Rora* ratio in different macrophage populations from WT mice. Data are shown as mean ± SEM. *p < 0.05 by 2-way ANOVA followed by Sidak’s multiple comparisons test. All statistical analyses were carried out using GraphPad Prism 8 for Windows (GraphPad Software). n = 3–6 mice per genotype except for ATM where each dot represents a pool of 3–4 mice. *NS* not significant, *AU* arbitrary unit. See also Fig. S5.

Contrasting with these results in 2 independent and complementary models of NAFLD, it was reported that RORα deletion in macrophages, achieved by using a similar strategy (Rora^fl/fl^ Lyz2^Cre^), predisposes mice to NASH after 12 weeks of HFD feeding^31^. NASH exacerbation was attributed to a key role of RORα in Kupffer Cell (KC) function^31^. To determine a possible cause for this discrepancy, we first assessed the extent of *Rora* exon 3 deletion in KC from chow diet fed mice. KC and other liver immune cell populations, sorted to a high level of purity (Fig. S5A–G), revealed efficient and specific deletion of *Ror*a exon 3 in KC (Fig. 5A). Interestingly, and similarly to publicly available transcriptomic data (Fig. S5H), we observed that KC expressed lower levels of *Rora* than other macrophage populations, with lung macrophages expressing the highest level (Fig. 5B).

Despite a significant *Ror*a exon 3 deletion, expression of the ROR target gene *Arntl* did not decrease in KC, unlike lung macrophages (Fig. 5C), splenic macrophages (Fig. 1F), BMDM (Fig. 1G) and ATM (Fig. 1H). The lack of effect of RORα deletion on *Arntl* expression in KC suggests a compensatory mechanism by another ROR protein. *Rorb* was not expressed in KC, similarly to other macrophage populations tested (data not shown), but *Rorc* expression was detected (Fig. 5D). No compensation of *Rorc* expression was found in any macrophage subsets from MKO mice compared to littermate controls. However, basal *Rorc* expression was between 5- and tenfold higher in KC compared to other macrophage subsets. The low *Rora* expression and the high *Rorc* expression in KC led to a *Rorc/Rora* ratio 10- to 50-fold higher than in other macrophage populations (Fig. 5E). Collectively, these results suggest that RORα deficiency is unlikely to induce major transcriptional effect in KC due to a concomitant high RORγ expression.

## Discussion

Over the past years, a considerable number of studies contributed to our understanding about the role of nuclear receptors, including RORs, in the regulation of lipid and glucose metabolism, circadian rhythm as well as the development and function of the immune system. Due to the pleiotropic effects and wide cell distribution of RORα and considering the key role of macrophages in metabolic diseases, we investigated the impact of RORα deletion in these cells on obesity, IR and NASH by using the LysM-Cre mice. Evaluation of deletion efficiency and decreased expression of a major RORα target gene in several myeloid subsets showed that macrophages were mostly impacted by RORα deletion in basal conditions although we cannot formally exclude that other myeloid subsets are also affected upon metabolic challenge.

Yet, importantly, we found no impact of LysM-Cre-mediated RORα deletion neither on HFD-induced obesity, IR and steatosis nor on CDAA diet-induced NASH. These findings contrast with an earlier report showing that LysM-Cre-mediated RORα deletion increases the susceptibility to HFD-induced NASH^31^, while both studies used the same HFD reference and duration of treatment. As feeding a HFD for 12 weeks is not per se leading to NASH, but rather to NAFL^9^, only a more pronounced steatosis (higher liver weight and triglyceride content) was reported in MKO mice in the earlier study^31^. Indicators of increased hepatic lipotoxicity such as higher plasma transaminase activity, increased expression of pro-inflammatory cytokines (*Tnf*, *Il1b* and *Il6*) and decreased expression of *Il10* in liver were also observed^31^. It was proposed that RORα directly regulates M2 polarization of KCs leading to increased IL-10 production which would protect hepatocytes against lipid accumulation^31^. While we also observed a tendency to lower *Il10* expression in livers from MKO mice upon HFD feeding (Fig. 3F) and a significant decrease upon CDAA diet feeding (Fig. 4F), we did not observe any exacerbation of steatosis or NASH. As the putative protective role for IL-10 was only based on in vitro findings, we believe that the reduced *Il10* expression in liver of MKO mice upon HFD is unlikely to contribute to aggravated steatosis. Further supporting this finding, IL-10-deficient mice fed a HFD for 12 weeks developed increased liver inflammation, but decreased steatosis and transaminase activity^32^, suggesting that beyond its well described anti-inflammatory function, endogenous IL-10 is not protective against HFD-induced steatosis.

Several factors might account for the discrepancy between our results and these earlier findings^31^. The microbiological status of the animal facility and housing conditions are two underestimated factors that might account for major differences in experimental outcome and it is not uncommon for this information to be missing. Specific pathogen free (SPF) status indicates that mice are free of defined pathogens including the mouse hepatitis virus and *Helicobacter hepaticus* which may interfere with liver function. Gut microbiota also impacts on NAFLD development^33^. The microbiota composition is highly variable between animal facilities, mouse lines and even between cages, especially when two genotypes are bred separately. Generation of littermate animals is critical to insure that both deficient and control mice harbor identical gut microbiota while post-weaning cohousing is ineffective^34^. Our mice were housed in SPF facility and bred to obtain WT and MKO littermate animals. No information was provided in the earlier study about the animal facility and littermate status.

Two additional factors that may also account for the observed discrepancies between studies are the floxed mice and the breeding strategy used. We generated mice with floxed *Rora* exon 3 while exon 4 was targeted in the earlier study^31^. Deletion of exon 4 may still result in the translation of a truncated protein containing a portion of the DBD that includes the exon 3-encoded zinc finger motif (Fig. S1C). This difference in deletion strategy is however unlikely to account for the observed phenotypic differences. The selected control animals represent a second major difference between the studies. The LysM-Cre mice carry an insertion of Cre recombinase into the *Lyz2* gene, leading to Cre expression under the control of the *Lyz2* promoter and enhancers, but abolishing endogenous *Lyz2* expression. While we intentionally maintained similar *Cre* and *Lyz2* expression between WT and MKO by using only hemizygous animals for this locus (comparing *Rora*^+*/*+^ *Lyz2*^*Cre/*+^ with *Rora*^*fl/fl*^* Lyz2*^*Cre/*+^), floxed mice (Rora^fl/fl^ Lyz2^+/+^) were used as WT control and compared with MKO mice missing information about the *Lyz2* locus (Rora^fl/fl^ Lyz2^Cre/?^) in the earlier study^31^. This latter strategy results in differential *Cre* and *Lyz2* expression between the WT, expressing no *Cre,* and the MKO mice expressing less or no *Lyz2*. Even though mammalian genomes possess no loxP sites, active Cre recombinase recognition sites, called pseudo loxP sites, have been reported^35^ and Cre expression mediates DNA damage, cell toxicity and apoptosis even in the absence of floxed alleles^36^. In addition to RORα deletion and exogenous Cre expression in macrophages, MKO mice generated with this strategy possess also at least one inactivated *Lyz2* allele and possibly a homozygous (whole body) *Lyz2* deletion. Indeed, based on the described genotyping strategy using a single PCR reaction to only analyze the Cre gene^31^, it is highly likely that MKO mice were actually also *Lyz2* deficient.

Lysozyme is an antimicrobial protein that catalyzes the hydrolysis of peptidoglycan between N-acetylglucosamine and N-acetylmuramic acid, contributing to the degradation of the Gram-positive bacterial cell wall. In humans, lysozyme is encoded by a single gene *LYZ*, whereas two genes exist in mouse, *Lyz1* and *Lyz2* encoding for lysozyme P and lysozyme M respectively. *Lyz2* is the most expressed lysozyme gene in mice with a high expression in the myeloid lineage, similar to the human *LYZ* gene. Expectedly, Lyz2-deficient mice develop more severe bacterial infections^37–39^. In addition to its antimicrobial properties, lysozyme is also an anti-inflammatory factor. Lysozyme inhibits serum complement activation^40^ and possesses a LPS-binding ability that reduces LPS-related inflammation^41^. Peptides derived from lysozyme cleavage inhibit production of pro-inflammatory cytokines, including TNF-α and IL-1β, by macrophages^42^. Moreover, lysozyme improves the antioxidant capacity of hepatocytes in vitro and in vivo, leading to a protection against oxidative stress in liver^43^. Lysozyme expression and activity is affected in various liver diseases^44–46^, but, to our knowledge, the role of *Lyz2* in NASH was never investigated. These roles in infection, inflammation and probably in liver homeostasis, suggest that *Lyz2* may play a role in NASH development. Thus, caution should be exerted when comparing mouse lines with different *Lyz2* copy numbers.

By using a 12-week HFD that induces liver steatosis (NAFL) or an 8-week CDAA diet that leads to NASH, we observed no differences between WT and MKO mice for any tested hepatic parameters including liver weight, triglyceride content, inflammatory and fibrosis gene expression, histology and transaminase activity. We also found no impact on obesity and glucose homeostasis. Investigations on KC showed that, despite an effective deletion of RORα, KCs were the only macrophage population without transcriptional effect on the ROR target gene *Arntl*. We found no compensatory expression of other ROR genes in MKO, but we evidenced a high level of *Rorc* expression specifically in KC that might offset the RORα deletion. The redundancy between ROR proteins is well established^17,47–49^. Hepatocytes express high levels of RORγ in addition to RORα^48^. It was shown that a single deletion of RORα or RORγ in hepatocytes affects only 2 and 6 transcripts respectively in whole liver transcriptome, while RORα/γ deletion leads to a broader effect with 299 genes affected, unambiguously demonstrating the redundancy between RORα and RORγ in hepatocytes^49^. Such a redundancy might be at play in KCs and further investigation with a double RORα/γ deficient mouse model might address this question. Moreover, usage of the new KC-specific Clec4f-Cre mouse line would improve the specificity towards KC and may result in a better deletion efficiency than the one reached with the LysM-cre mice^50^.

In conclusion RORα deletion in macrophages using the LyzM-Cre system has no impact on the development of obesity, IR and NASH. We suggest that the previously reported impact of RORα deletion in macrophages on NASH^31^ likely does not result from a specific effect of RORα deletion, but rather from a different *Cre* or *Lyz2* copy number between WT and MKO.

## Methods

### Generation of RORα MKO Mice

We generated mice harboring a floxed allele of *Rora* (*Rora*^*fl/fl*^) by flanking exon 3 with two *loxP* sequences. The strategy and outcome on RORα1 and RORα4 are summarized in figures S1A and S1B respectively. Briefly, a targeting vector containing loxP sites, a FRT-floxed neomycin cassette and homologous regions surrounding exon 3 was constructed and transfected in embryonic stem (ES) cells derived from 129/Sv. After screening for homologous recombination by southern blot and PCR, ES cells were injected into C57BL/6 blastocysts. Neomycin cassette was removed in vivo by using FLP deleter mice in C57BL/6 background. Finally, mice were backcrossed with C57BL/6 J mice for at least six generations.

To achieve the deletion of RORα in macrophages, *Rora*^*fl/fl*^ mice were crossed with LysM-Cre transgenic mice (Jackson laboratory) in which Cre recombinase is expressed under the control of endogenous *Lyz2* promoter. Littermates RORα-deficient (MKO, *Rora*^*fl/fl*^* Lyz2*^*Cre/*+^) and WT (*Rora*^+*/*+^ *Lyz2*^*Cre/*+^) mice were generated by crossing *Rora*^*fl/*+^ *Lyz2*^*Cre/Cre*^ with *Rora*^*fl/*+^ *Lyz2*^+*/*+^ mice. All mice were genotyped twice.

### Mouse studies

Mice were kept on a 12-h light/dark cycle in the SPF animal facility from the Institut Pasteur de Lille with ad libitum access to food and water. Littermate WT and MKO mice were maintained all along the experiment procedures in 904 cm^2^ cages (Green line GR900, Tecniplast) with 6–12 mice per cage and a ratio WT:MKO tending to 1:1. Only male mice were used for the experiments. All animal procedures were approved by the ethical committee for animal experimentation of the Nord-Pas-de-Calais Region (CEEA75) (APAFIS#7160-2017040313471173) in accordance with European guidelines on the protection of animals used for scientific purposes (2010/63/UE).

Ten-week-old RORα WT and their littermates RORα MKO mice were fed with a 60% high-fat diet (HFD, Research Diet, D12492) for 12 weeks or maintained under chow diet (SAFE, #A04). To induce a NASH-like disease, ten-week-old RORα WT and MKO mice were fed with a choline-deficient, L-amino acid-defined (CDAA) diet with 35% sucrose, 21% fat and 2% cholesterol (Ssniff, custom diet) for 8 weeks. In addition to CDAA diet, mice also received monosaccharides in the drinking water (42 g/L, fructose:glucose ratio of 55:45). Weight of mice was measured weekly. Before sacrifice, mice were fasted for 5 h. Mice were sacrificed at ZT3 (10 am) for HFD and at ZT7 (2 pm) for CDAA experiment.

### Mouse genotyping

DNA was extracted from tail with REDExtract-N-Amp Tissue PCR Kit (Sigma, #XNAT-1000RXN). Floxed *Rora* was detected by PCR with the *Rora* genotyping primers (Supplementary Table 1) and the following cycling conditions: 1 cycle at 94 °C for 3 min; 35 cycles at 94 °C for 30 s, 55 °C for 30 s, 72 °C for 1 min; and 1 cycle at 72 °C for 10 min; hold at 4 °C. Samples were separated by gel electrophoresis on a 1.5% agarose gel. *Rora*^+*/*+^ gave a single band at 250 bp, *Rora*^*fl/fl*^ at 340 bp and *Rora*^*fl/*+^ had both bands. Endogenous *Lyz2* and *Cre* were detected with the *Lyz2* genotyping and *Cre* genotyping primers respectively (Supplementary Table 1) and the following cycling conditions: 1 cycle at 94 °C for 3 min; 35 cycles at 94 °C for 1 min, 63 °C for 1 min, 72 °C for 90 s; and 1 cycle at 72 °C for 10 min; hold at 4 °C. Samples were separated by gel electrophoresis on a 1.5% agarose gel. *Lyz2*^+/+^ gave a single band at 350 bp for *Lyz2* PCR while *Lyz2*^Cre/Cre^ gave a single band at 700 bp for *Cre* PCR. *Lyz2*^Cre/+^ gave bands for both *Lyz2* and *Cre* PCR. Agarose gels were acquired with a Gel Doc XR system (Bio-Rad) and the Image Lab software verion 2.0 build 8 for PC (Bio-Rad, https://www.bio-rad.com/fr-fr/product/image-lab-software?ID=KRE6P5E8Z). The following acquisition settings were chosen: application: SYBR Safe; Image exposure: automatically optimized.

### Bone marrow–derived macrophages

Bone marrow was isolated from tibia and femur of mice. Bone marrow cells were culture in RPMI 1640 with Hepes and L-glutamine supplemented with 10% fetal bovine serum, 20% L929-conditioned medium and 25 µg/mL gentamycin for one week. After three days of culture, fresh medium was added. After one week of culture, supernatant was discarded and adherent bone marrow–derived macrophages (BMDMs) were washed two times with PBS. BMDMs were collected by using a cell scraper, counted and plated at a concentration of 10^6^ cells/ml. After 24 h, BMDMs were treated with 100 nM dexamethasone (Sigma, #D1756) for 2 h to synchronize cells, washed and maintained in complete medium for 32 h to reach the pic of RORα activity corresponding to ZT0 in vivo.

### Insulin and glucose tolerance tests

After 10 weeks of HFD, a Glucose Tolerance Test (GTT) was performed by intraperitoneal injection of glucose (1 g/kg). Tail blood sample was collected before glucose injection to measure fasting insulin by ELISA (Mercodia #10-1247-10) according to manufacturer instruction. After 11 weeks of HFD, an Insulin Tolerance Test (ITT) was performed by intraperitoneal injection of human insulin (1 IU/kg) (Actrapid, Novo Nordisk). Glycemia was measured from tail before and 15, 30, 60, 90 and 120 min after glucose or insulin injection by using a glucose meter (Accu-Check performa, Roche). Before GTT and ITT, mice were fasted for 5 h at ZT2 (9 am) and the tests were performed at ZT7 (2 pm).

### Cell sorting

Chow diet fed mice were sacrificed at ZT0 (7 am) and tissue kept in PBS on ice until processing. For epididymal adipose tissue, between 3 and 4 mice were pooled. Spleen was gently pressed on a 70 µm cell strainer by using a 1 mL syringe plunger. Adipose tissue, liver and lung were minced with scissors and digested in RPMI containing 1 mg/mL collagenase D (Roche # 11088882001) at 37 °C under agitation for 30, 45 and 60 min respectively and then carefully passed several times through an 18 or 19 G needle to obtain a single cell suspension. Red blood cells were lysed with ammonium chloride-based buffer and cells were blocked by using a combination of anti-CD16/CD32 (clone 2.4G2) (BD Biosciences, #553142) and anti-FcγRIV (clone 9E9) (BioLegend, #149502). Antibodies used for staining are provided in Supplementary Table 2. Cell suspension was directly run into an Influx sorter (Becton Dickinson) (Plateau d’Immunophenotypage Metabolique, Inserm U1011) equipped with an 86 µm nozzle and tuned at a pressure of 24.7 psi and a frequency of 48.25 kHz. Sample fluid pressure was adjusted to reach an event rate of maximum 10 000 events/sec. In spleen, cells of interest were selected in the following order: T cells (CD45^+^ CD3ε^+^ TCRβ^+^ MHC-II^−^), B cells (CD45^+^ CD19^+^ MHC-II^+^), Neutrophils (CD45^+^ CD3ε^−^ TCRβ^−^ CD19^−^ CD11b^+^ Ly6G^+^), Macrophages (CD45^+^ CD3ε^−^ TCRβ^−^ CD19^−^ Ly6G^−^ F4/80^+^), monocytes (CD45^+^ CD3ε^−^ TCRβ^−^ CD19^−^ Ly6G^−^ F4/80^−^ CD11b^+^ CD115^+^) and DCs (CD45^+^ CD3ε^−^ TCRβ^−^ CD19^−^ Ly6G^−^ F4/80^−^ CD115^−^ CD11c^+^ MHC-II^+^). In liver, cells of interest were selected in the following order: T cells (CD45^+^ CD3ε^+^ TCRβ^+^ CD19^−^ CD20^−^), B cells (CD45^+^ CD19^+^ CD20^+^ MHC-II^+^), Kupffer cells (CD45^+^ CD3ε^−^ TCRβ^−^ CD19^−^ CD20^−^ F4/80^+^ Clec4F^+^) and other (CD45^+^ CD3ε^−^ TCRβ^−^ CD19^−^ CD20^−^ F4/80^−^ Clec4F^−^). Adipose tissue macrophages were selected as CD45^+^ CD3ε^−^ TCRβ^−^ CD19^−^ CD20^−^ F4/80^+^ CD64^+^ and lung macrophages as CD45^+^ CD3ε^−^ TCRβ^−^ CD19^−^ F4/80^+^ CD64^+^ CD11b^low^ SiglecF^+^.

For bone marrow (BM) neutrophils, BM was first isolated from tibia and femur of mice. Red blood cells were lysed with ammonium chloride-based buffer and BM cells were blocked by using a combination of anti-CD16/CD32 (clone 2.4G2) (BD Biosciences, #553142) and anti-FcγRIV (clone 9E9) (BioLegend, #149502). Cells were incubated with PE rat anti-mouse Ly-6G (Clone 1A8) (BD Biosciences, #551461) for 30 min and purified by magnetic separation with anti-PE MicroBeads (Miltenyi Biotec, #130-048-801) according to the manufacturer’s instructions.

### Histology

The median lobes of liver were collected and fixed in 4% paraformaldehyde for 48 to 72 h. Tissues were embedded in paraffin by using a STP 120 Spin Tissue Processor (Microm Microtech) and an EG1160 Tissue Embedding Station (Leica). Paraffin-embedded samples were cut at a thickness of 3 μm and sections were transferred on a gelatin-coated slide for hematoxylin and eosin (H&E) staining and on a Superfrost Plus slides (Thermo Scientific, # J1800AMNZ) for Sirius Red staining. Staining was performed with a Leica autostainer XL and the following steps for H&E staining: xylene (2 min), xylene (2 min), 100% ethanol (2 min), tap water (2 min), hematoxylin (Sigma, #HHS128) (3 min), tap water (2 min), 70% ethanol 0.25% HCl (6 s), tap water (2 min), 90% ethanol (2 min), eosin (Sigma, #HT1101128) (2 min), 90% ethanol (6 s), 100% ethanol (1 min) and finished in xylene before mounting with Mercoglas (Merck, #103973). For Sirius red staining, the process was the following: xylene (2 min), xylene (2 min), 100% ethanol (2 min), tap water (2 min), 0.1% Direct Red 80 (Sigma, #365548) in saturated picric aqueous solution (60 min), tap water (8 min), 100% ethanol (1 min) and finished in xylene before mounting with Mercoglas. Images were acquired on an Eclipse Ti-U microscope (Nikon) and quantified with Image J version 1.51j8 software (NIH, https://imagej.nih.gov/ij/).

### Metabolic parameters

Before sacrifice and after 5 h of fasting, blood samples were collected from the retro orbital sinus of mice. Plasma alanine aminotransferase (ALAT), aspartate aminotransferase (ASAT), total cholesterol and triglycerides were measured on a Konelab 20 (Thermo Fisher) with reagents from Thermo Scientific for ALAT (#981769) and ASAT (#981771) and reagents from DiaSys for cholesterol (#113009910026) and triglycerides (#157109910026). The plasma ALAT and ASAT measurement under CDAA diet was performed without fasting for the time point T0, 2, 4 and 6 weeks.

### Measurement of liver triglycerides

Lipids were extracted from the liver caudate lobe. A weighted piece of tissue was homogenized with T10 Ultra-Turrax (Ika) in PBS 1% Triton. Samples were transferred into glass tubes and mixed with a 2:1 chlorofrom:methanol mixture. After centrifugation, upper- and inter-phase were discarded. The lower organic phase was evaporated under nitrogen flow and reconstituted in 1% Triton X100. Triglyceride content was measured with Triglycerides FS kit (DiaSys, #157109910026).

### RT-qPCR

Total RNA was extracted from the snap-frozen tissues or sorted cell pellets by using TRIzol reagent (Ambion, #15596018) according to the manufacturer’s instructions. DNase treatment was performed (Thermo Scientific, #EN0521) and RNA was reverse-transcribed to complementary DNA (cDNA) by using the high capacity cDNA reverse transcription kit (Applied Biosystems, #4368813). qPCR was performed by using Brilliant II SYBR Green QPCR Master Mix (Agilent, #600828) and ran on a Mx3000P qPCR system (Agilent). Relative changes in mRNA expression (Fold change, FC) were calculated by the 2^−ΔΔCT^ method. Absolute changes in mRNA expression were calculated with the formula 2^−ΔCT^ × 100 and expressed as arbitrary unit (AU). The mean of four housekeeping genes (*Rplp0*, *Ppia*, *Rpl4* and *Rps29*) was used to normalize mRNA expression. The primers used for qRT-PCR are listed in Supplementary Table 1.

### Western blotting

Tissues were weighted and homogenized with a T10 Ultra-Turrax (Ika) in protect buffer (water with cOmplete protease inhibitor cocktail (Roche, #11836145001) and PhosSTOP (Roche, #04906837001)) (3.75 µL/mg for adipose tissue and 7.5 µL/mg for muscle and liver). Next, total protein lysates were mixed with 4X Laemmli buffer (Tris–HCl 250 mM pH 6.8, 40% glycerol, 8% SDS, 12% β-mercaptoethanol and bromophenol blue) and heated at 100 °C for 5 min. Total protein extracts were subjected to SDS-PAGE (10% polyacrylamide gel) and transferred on a 0.22 µm nitrocellulose membrane (Li-Cor, #P/N 926-31092). After blocking in 5% BSA, membranes were probed with mouse anti-Akt (Cell Signaling, #2920) and rabbit anti-phospho-Akt (Ser473) (Cell Signaling, #4058) monoclonal antibodies. The secondary antibodies used for the revelation were Alexa Fluor 680 anti-mouse IgG (Jackson ImmunoResearch, #715-625-150) and Alexa Fluor 790 anti-rabbit IgG (Jackson ImmunoResearch, #711-655-152). Immunoblots were scanned with an Odyssey CLx Imaging System (LI-COR) and with the Image studio Version 4.0.21 software (LI-COR, https://www.licor.com/bio/image-studio/). The following acquisition settings were chosen: setup: Western; acquisition channels: auto; scan resolution: 169 µm; scan quality: low; focus offset: 0.0 mm. Images were quantified with the Image studio Lite Version 4.0.21 software (LI-COR, https://www.licor.com/bio/image-studio-lite/).

### Statistical analyses

All statistical analyses were carried out using GraphPad Prism 8 for Windows (GraphPad Software) and presented as means ± SEM. The study was done blinded for genotype. Only one mouse (WT) was excluded from the analysis because it did not reach an appropriate weight gain under HFD feeding (Body weight: 31.2 g; ingAT: 0.165 g; epiAT: 0.348 g). No mice were excluded in CDAA diet group. Data were analyzed with 2-way ANOVA and Sidak’s multiple comparisons post-hoc test or Student's *t*-test. Values with *P* < *0.05* were considered as significant. All statistical details including statistical test used and exact value of n are described in each figure legend. Each data point represents genuine replication, also called true replicate, and were obtained from a single measurement or multiple measurements illustrated by the mean, such as gene expression by RT-qPCR that was assessed in duplicate or histology quantification made on five random fields per mice. For sake of clarity, results of statistical comparisons between WT and MKO mice upon Chow feeding were not illustrated on the figures but were not significant for all the parameters. In Figs. 2, 3, and 4, the diet effect was analyzed by 2-way ANOVA and was significant for all the parameters with the exception of liver weight (Fig. 3B) that was not statistically significant between chow and HFD (*p* = 0.06). The datasets used in this study are available from GEO under the accession number GSE104342^51^ and GSE56682^52^ and from data assembled by the ImmGen consortium (http://www.immgen.org/)^53^.

## Supplementary information

Supplementary information.

## Data Availability

Further information and requests for resources and reagents should be directed to and will be fulfilled by David Dombrowicz (david.dombrowicz@inserm.fr).
