# Supplementary material for: Deletion of the nuclear receptor RORα in macrophages does not modify the development of obesity, insulin resistance and NASH
Source: Sci Rep. 2020 Dec 3;10:21095. doi: 10.1038/s41598-020-77858-6 (PMC7713245; doi:10.1038/s41598-020-77858-6)
Supplement: Supplementary file 1 — Supplementary information. [file 41598_2020_77858_MOESM1_ESM.pdf]

Deletion of the nuclear receptor RORα in macrophages does not modify the development of obesity, insulin resistance and NASH

Laurent L'homme[1,2], Benan Pelin Sermikli[1,2], Olivier Molendi-Coste[1,2], Sébastien Fleury[1], Sandrine Quemener[1], Mathilde Le Maître[1], Marie-Laure Joseph[1], Laurent Pineau[1], Christian Duhem[1], Barbara Gross[1], Emmanuelle Vallez[1], Anne Tailleux[1], Bart Staels[1,3], David Dombrowicz[1,3,*]

[1]Univ. Lille, Inserm, CHU Lille, Institut Pasteur de Lille, U1011-EGID, F-59000 Lille, France

[2]These authors contributed equally

[3]These authors shared senior authorship

*Correspondence : david.dombrowicz@pasteur-lille.fr

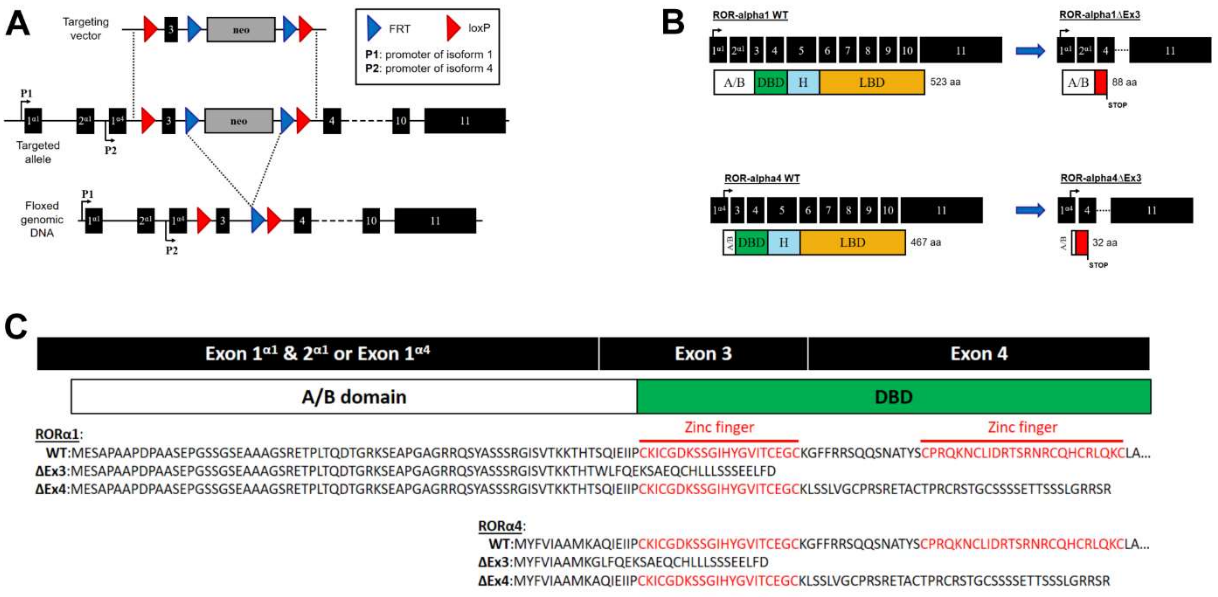

Figure S1: Characterization of RORα MKO mice (Related to Figure 1).

(A) Schematic representation of the strategy to target exon 3 of *Rora*. **(B)** mRNA structures of RORα1 and RORα4 and the resulting proteins in both WT and MKO (ΔEx3) mice. Exons are represented in black. Protein predictions are illustrated below mRNA and functional domains are indicated with colors. **(C)** Comparison of *Rora* exon 3 and exon 4 deletion on protein translation. Zinc finger motifs are indicated in red. Protein predictions were generated with Translate tool from ExPASy (https://web.expasy.org/translate) by using GenBank sequences for RORα1 (NM_013646.2) and RORα4 (NM_001289916.1).

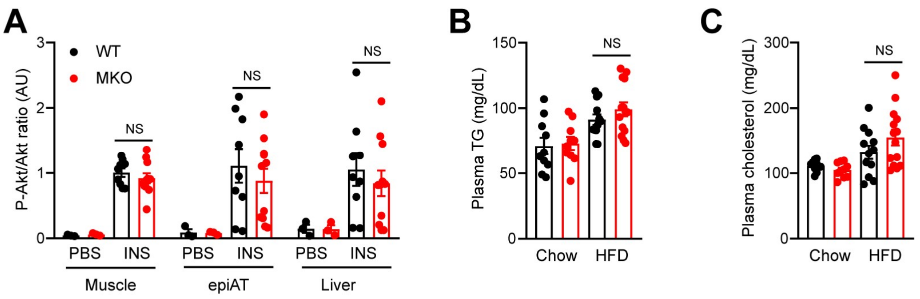

<u>Figure S2</u>: Effect of RORα deletion in macrophages on HFD-induced obesity and IR (Related to Figure 2).

Ten weeks old WT and MKO mice were fed with either a chow or a HFD for 12 weeks. **(A)** Quantification of western blot for phospho-Akt in skeletal muscle, epididymal adipose tissue (epiAT) and liver. Quantification were performed with the Image studio Lite Version 4.0.21 software (LI-COR, https://www.licor.com/bio/image-studio-lite/) **(B)** Plasma triglyceride (TG). **(C)** Plasma cholesterol. Data are shown as mean ± SEM. 2-way ANOVA followed by Sidak's multiple comparisons test was performed. All statistical analyses were carried out using GraphPad Prism 8 for Windows (GraphPad Software). n=10-15 mice per group. NS: Not significant; AU: Arbitrary unit.

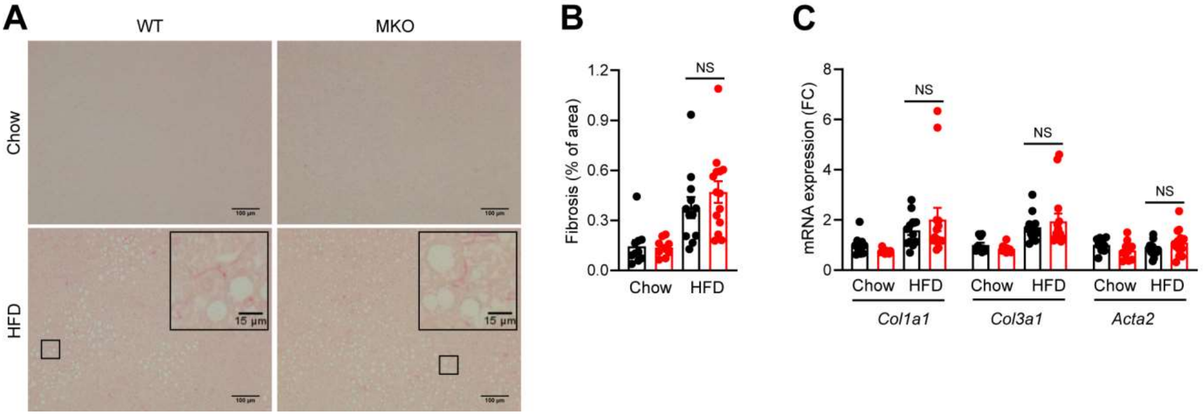

Figure S3: Effect of RORα deletion in macrophages on HFD-induced fibrosis (Related to Figure 3).

Ten weeks old WT and MKO mice were fed with either a chow or a HFD for 12 weeks (**A-B**) Sirius red staining of liver sections (**A**) and quantification of fibrosis (**B**). Images were acquired on an Eclipse Ti-U microscope (Nikon) and quantified with Image J version 1.51j8 software (NIH, https://imagej.nih.gov/ij/). **(C)** mRNA expression levels measured by RT-qPCR for *Col1a1, Col3a1, and Acta2* genes in the liver. Data are shown as mean ± SEM. 2-way ANOVA followed by Sidak's multiple comparisons test was performed. All statistical analyses were carried out using GraphPad Prism 8 for Windows (GraphPad Software). n=10-15 mice per group. NS: Not significant; FC: Fold Change

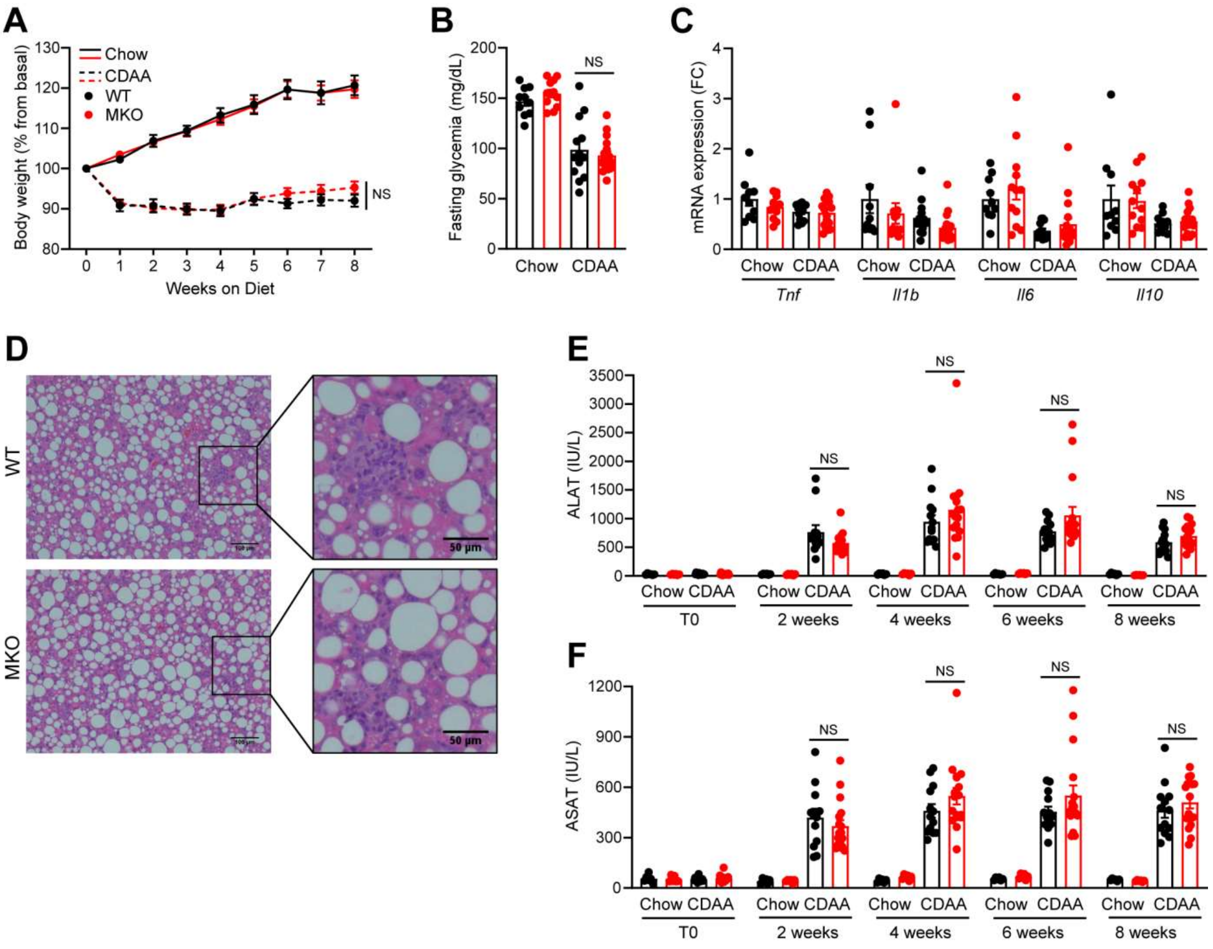

<u>Figure S4:</u> Effect of RORα deletion in macrophages upon CDAA diet feeding (Related to Figure 4).

Ten weeks old WT and MKO mice were fed with either a chow or a CDAA diet for 8 weeks. **(A)** Body weight gain. **(B)** Fasting glycemia. **(C)** mRNA expression levels measured by RT-qPCR for *Tnf, Il1b, Il6, and Il10* genes in epiAT. **(D)** Hematoxylin & Eosin staining of liver sections. Images were acquired on an Eclipse Ti-U microscope (Nikon) and prepared with Image J version 1.51j8 software (NIH, https://imagej.nih.gov/ij/). **(E-F)** Kinetics of plasma transaminases activity. Data are shown as mean ± SEM. 2-way ANOVA followed by Sidak's multiple comparisons test was performed. All statistical analyses were carried out using GraphPad Prism 8 for Windows (GraphPad Software). n=10-17 mice per group. NS: Not significant; FC: Fold Change.

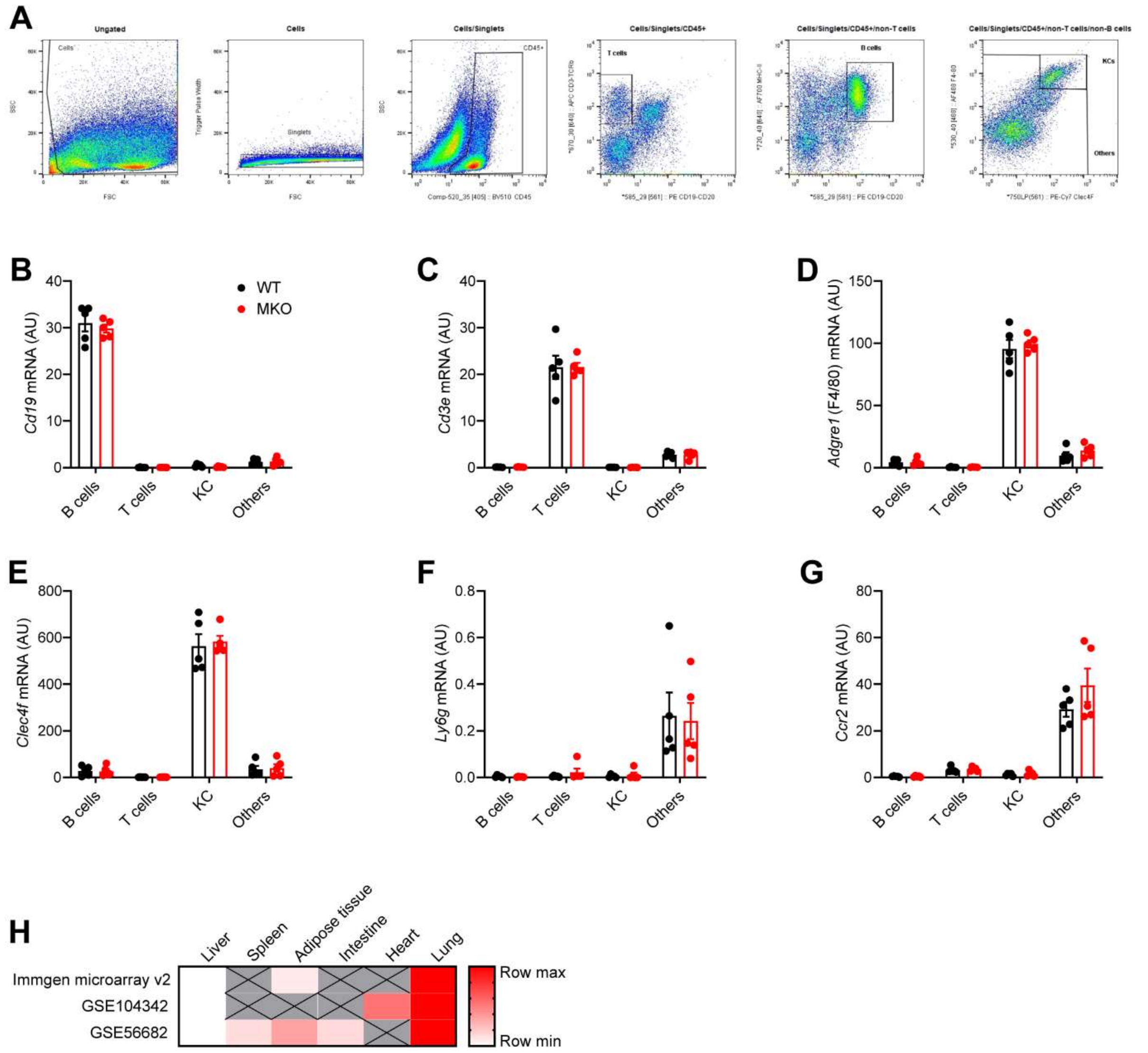

<u>Figure S5:</u> Quality control of Kupffer Cells (KCs) sorting (Related to Figure 5).

(**A**) Representative gating strategy for cell sorting of T cells, B cells, KCs and other cells ($CD45^+$ non-T cells, -B cells and -KCs) from liver. (**B-G**) mRNA expression levels measured by RT-qPCR for the *Cd19* **(B),** *Cd3e* **(C),** *Adgre1* **(D),** *Clec4f* **(E),** *Ly6g* **(F)** and *Ccr2* (**G**) genes in B cells, T cells, KCs and Others sorted from the liver of WT and MKO mice. (**H**) Heatmap of *Rora* expression in different macrophage populations from public databases. Data are shown as mean ± SEM. n=5 mice per genotype. AU: Arbitrary unit.

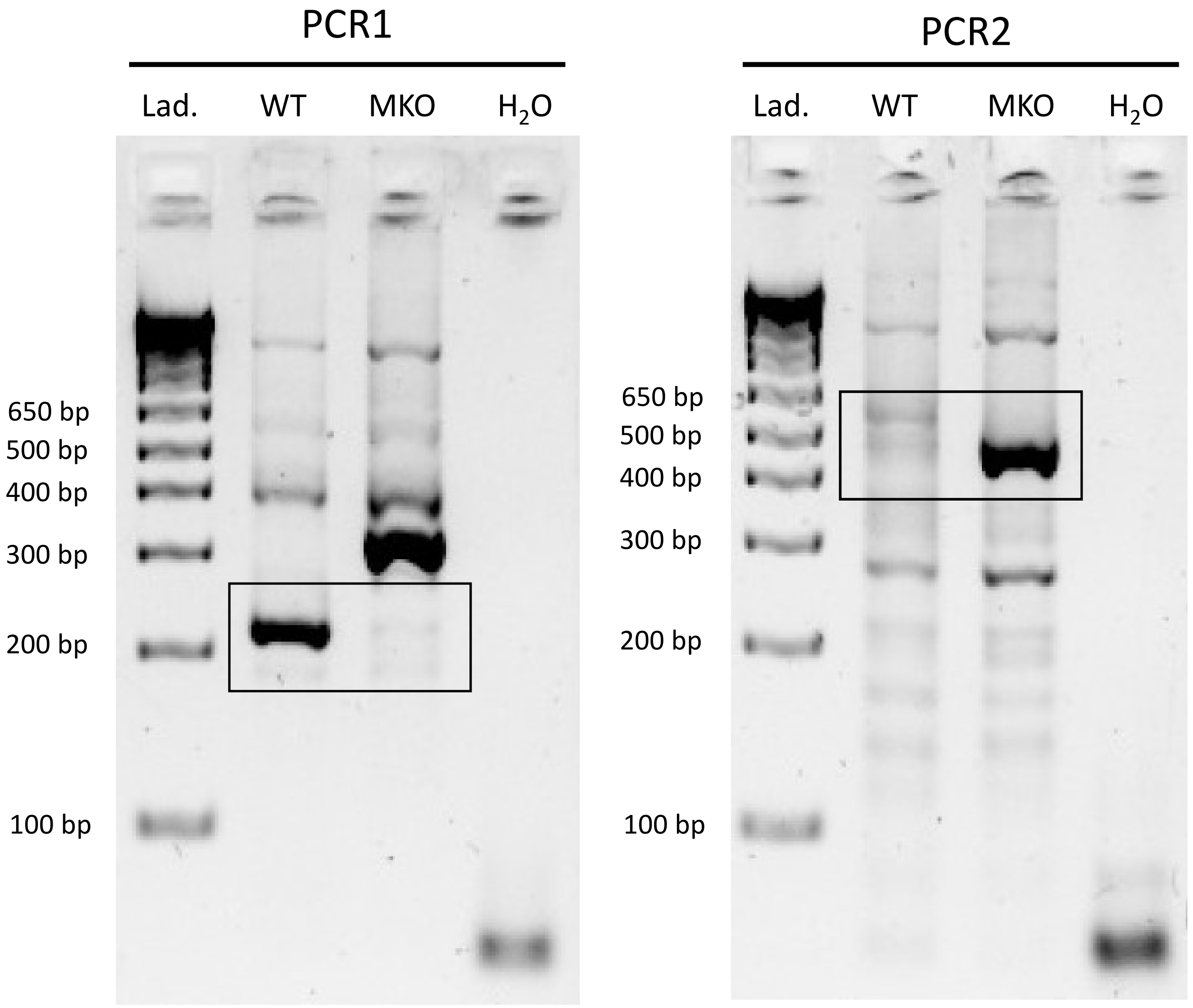

Figure S6: Uncropped agarose gel (related to Fig. 1B)

Lad., DNA Ladder.

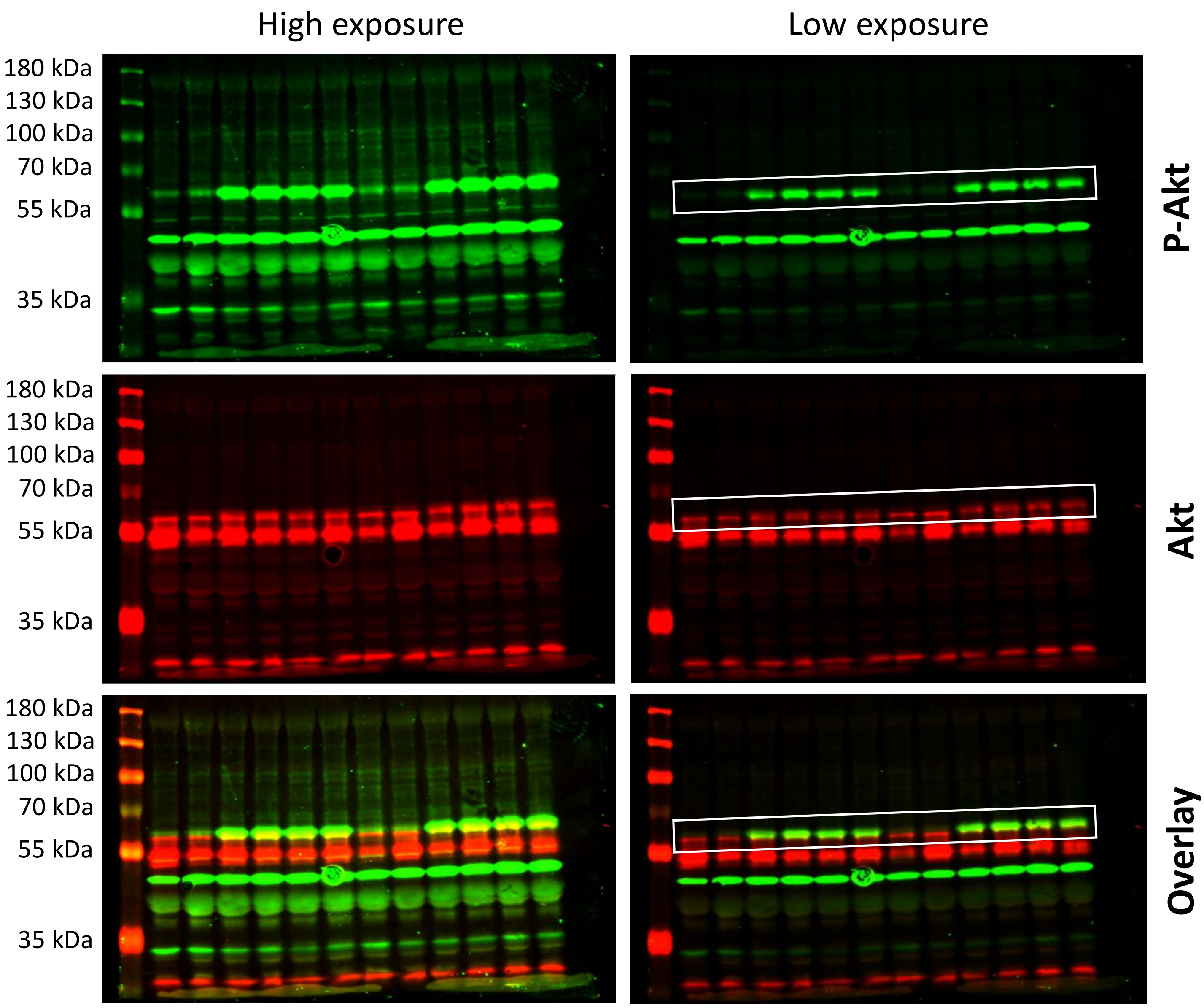

Figure S7: Uncropped WB images for muscle (related to Fig. 2H)

Akt molecular weight: 60kDa

Rabbit monoclonal anti-phospho-Akt (Ser473) (Clone 193H12) (Cell Signaling, #4058)

Mouse monoclonal anti-Akt (Pan) (Clone 40D4) (Cell Signaling, #2920)

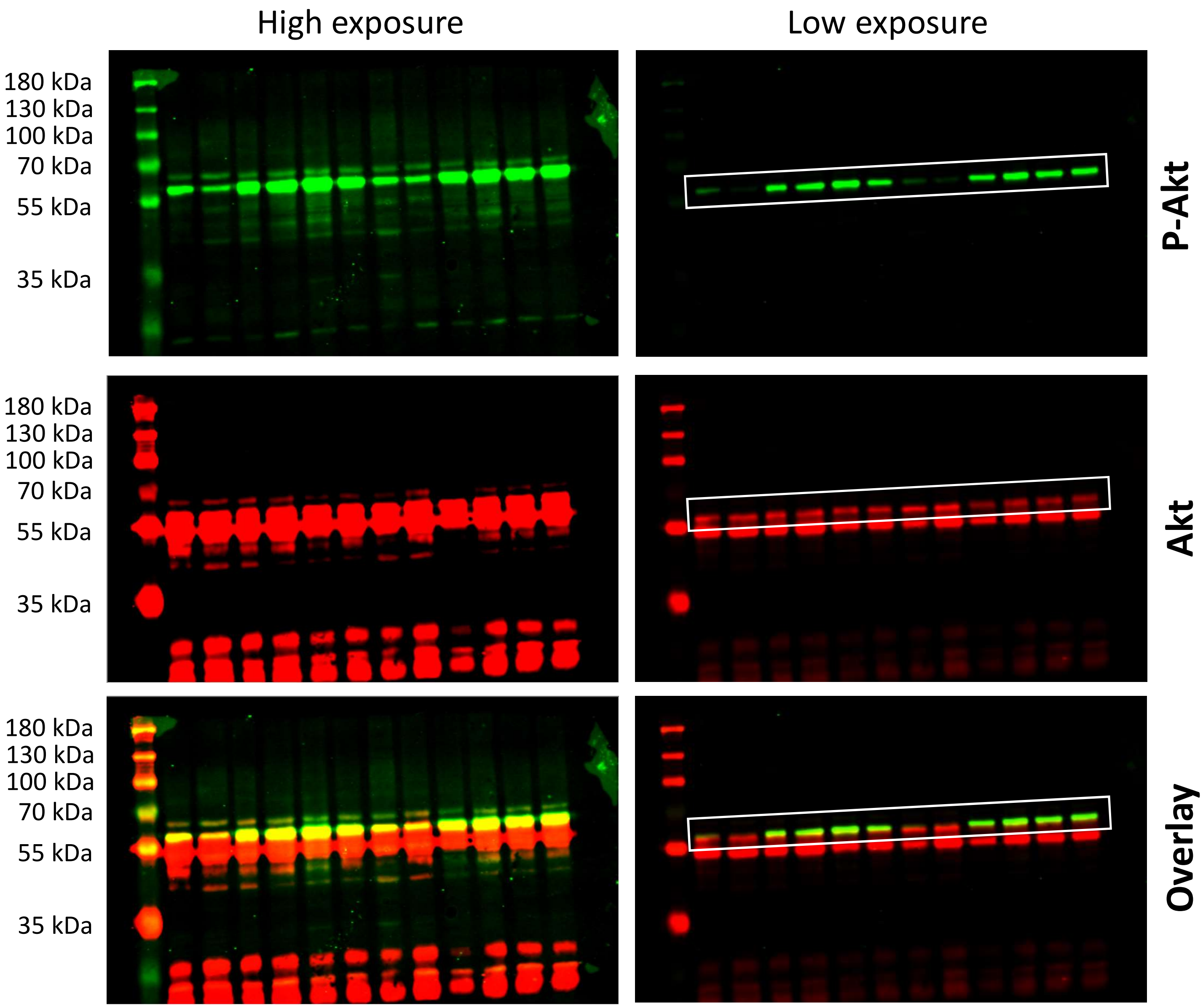

Figure S8: Uncropped WB images for epiAT (related to Fig. 2H)

Akt molecular weight: 60kDa

Rabbit monoclonal anti-phospho-Akt (Ser473) (Clone 193H12) (Cell Signaling, #4058)

Mouse monoclonal anti-Akt (Pan) (Clone 40D4) (Cell Signaling, #2920)

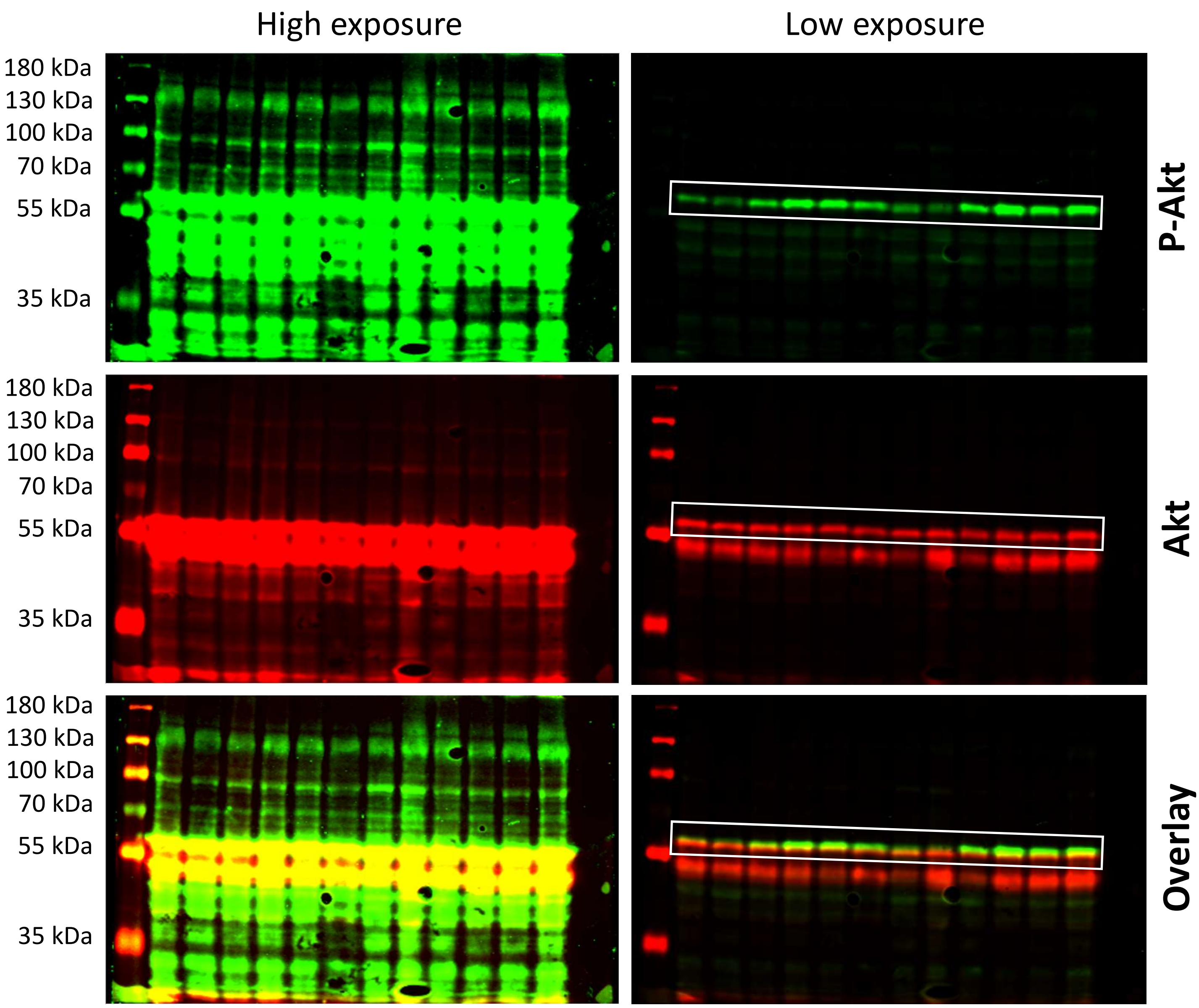

Figure S9: Uncropped WB images for liver (related to Fig. 2H)

Akt molecular weight: 60kDa

Rabbit monoclonal anti-phospho-Akt (Ser473) (Clone 193H12) (Cell Signaling, #4058)

Mouse monoclonal anti-Akt (Pan) (Clone 40D4) (Cell Signaling, #2920)

Supplementary Table 1 : Primers

	Forward	Reverse
**Genotyping**		
Rora (PCR1)	CATGCATACACCCACACACATCACAACC	CTTGATGCACTGATGTAATAACATATCC
Lyz2	CTTGGGCTGCCAGAATTTCTC	TTACAGTCGGCCAGGCTGAC
Cre	CTTGGGCTGCCAGAATTTCTC	CCCAGAAATGCCAGATTACG
**Excision**		
Rora (PCR2)	CATGCATACACCCACACACATCACAACC	CAGCCGTGTGAAACTTACGGGAAATACC
**qPCR**		
Rplp0	GGGAAGGTGTAATCAGTCTCCACAG	CATGCTGAACATCTCCCCCTTCTCC
Ppia	GCATACGGGTCCTGGCATCTTGTCC	ATGGTGATCTTCTTGCTGGTCTTGC
Rpl4	CTGAACCCTTACGCCAAGAC	CCTTCTCGGATTTGGTTGCC
Rps29	GATCCGCAAATACGGGCTG	CCATTCAAGGTCGCTTAGTCC
Rora Exon 3	TTCCATGCAAGATCTGTGGAGAC	TGCAGCCTTCACACGTAAT
Rorb	CCCACACCTACGAGGAAATCAA	CATGAAGCCTGTTATCCGCTTG
Rorc	GGTACCATATGCCTCTCTGA	ATCTCCCACATTGACTTCCT
Lyz2	CAATGTGCAAAGAGGGTGGTG	TAGAGGGGAAATCGAGGGAATG
Cre	CCTGGAAAATGCTTCTGTCCG	CAGGGTCTTATAAGCAATCCC
Arntl	GGACTTCGCCTCTACCTGTTC	ACCCGTATTTCCCCGTTC
Ob	GGTGTGAAAGAACCTGAGCTGAGG	CAGTGGATGCTAATGTGCCCTG
Tnf	AGCACAGAAAGCATGATCCG	CCCGAAGTTCAGTAGACAGAAGAG
Il1b	GCCACCTTTTGACAGTGATGAG	CCTGAAGCTCTTGTTGATGTGC
Il6	CCAGTTGCCTTCTTGGGACTG	CAGGTCTGTTGGGAGTGGTATCC
Il10	AGGCGCTGTCATCGATTTCTC	TGGCCTTGTAGACACCTTGGTC
Col1a1	AGGCATAAAGGGTCATCGTGG	GAGACCGTTGAGTCCGTCTTT
Col3a1	TGGCAATGTAAAGAAGTCTC	CCCAGTGTGTTTAGTACAGC
Acta2	GCCGAGATCTCACCGACTAC	CAATCTCACGCTCGGCAGTA
Cd19	ACCTGACCATCGAGAGGCACGTG	CCTGGCGGGGTCAGTCATTCGCTT
Cd3e	ACTGGAGCAAGAATAGGAAG	GGATGGGCTCATAGTCTG
Adgre1	CTTTGGCTATGGGCTTCCAGTC	GCAAGGAGGACAGAGTTTATCGTG
Clec4f	CCCAGGACCCTGCGGCACGTC	CCGAAGCAGGAGGGAGGGAGGCTC
Ly6g	TGATGGATTTTGCGTTGCTCTG	GTATTGTCCAGAGTAGTGGGGC
Ccr2	ATTCTCCACACCCTGTTTCG	CATGGCCTGGTCTAAGTGCT

Supplementary Table 2 : Antibodies for cell sorting

	Source	Identifier
**Spleen**		
BV510Anti-CD45 (Clone 30-F11)	BioLegend	#103137
PE-CF594 Anti-CD3e (Clone 145-2C11)	BD Biosciences	#562286
PE-CF594 Anti-TCR β Chain (Clone H57-597)	BD Biosciences	#562841
PE-CF594 Anti-CD19 (Clone 1D3)	BD Biosciences	#562291
BUV395 Anti-CD11b (Clone M1/70)	BD Biosciences	#563553
BV421 Anti-Ly-6G (Clone 1A8)	BioLegend	#127627
PE/Cy7 Anti-F4/80 (Clone BM8)	BioLegend	#123113
FITC Anti-I-A/I-E (MHC class II) (Clone M5/114.15.2)	BioLegend	#107605
PE Anti-CD115 (CSF-1R) (Clone AFS98)	BioLegend	#135505
APC/Cy7 Anti-CD11c (Clone N418)	BioLegend	#117323
**Liver**		
BV510 Anti-CD45 (Clone 30-F11)	BioLegend	#103137
APC Anti-CD3e (Clone 145-2C11)	BD Biosciences	#553066
APC Anti-TCR β Chain (Clone H57-597)	BD Biosciences	#553174
PE Anti-CD19 (Clone 1D3)	BD Biosciences	#557399
PE Anti-CD20 (Clone AISB12)	eBioscience	#12-0201-82
AF700 Anti-I-A/I-E (MHC class II) (Clone M5/114.15.2)	BioLegend	#107622
AF488 Anti-F4/80 (Clone BM8)	BioLegend	#123120
Anti-CLEC4F/CLECSF13 (Clone 370901)*	Bio-Techne	#MAB2784
**Adipose tissue**		
BV510 Anti-CD45 (Clone 30-F11)	BioLegend	#103137
APC Anti-CD3e (Clone 145-2C11)	BD Biosciences	#553066
APC Anti-TCR β Chain (Clone H57-597)	BD Biosciences	#553174
PE Anti-CD19 (Clone 1D3)	BD Biosciences	#557399
PE Anti-CD20 (Clone AISB12)	eBioscience	#12-0201-82
AF700 Anti-I-A/I-E (MHC class II) (Clone M5/114.15.2)	BioLegend	#107622
AF488 Anti-F4/80 (Clone BM8)	BioLegend	#123120
BV711 Anti-CD64 (FcγRI) (Clone X54-5/7.1)	BioLegend	#139311
**Lung**		
BV510 Anti-CD45 (Clone 30-F11)	BioLegend	#103137
PE-CF594 Anti-CD3e (Clone 145-2C11)	BD Biosciences	#562286
PE-CF594 Anti-TCR β Chain (Clone H57-597)	BD Biosciences	#562841
PE-CF594 Anti-CD19 (Clone 1D3)	BD Biosciences	#562291
PE/Cy7 Anti-F4/80 (Clone BM8)	BioLegend	#123114
BV711 Anti-CD64 (FcγRI) (Clone X54-5/7.1)	BioLegend	#139311
BUV395 Anti-CD11b (Clone M1/70)	BD Biosciences	#563553
AF647 Anti-Siglec-F (Clone E50-2440)	BD Biosciences	#562680

* Anti-CLEC4F antibody was coupled with PE/Cy7 by using PE/Cy7 Conjugation Kit - Lightning-Link (Abcam, #ab102903).
